# Unveiling senescence-associated secretory phenotype in epidermal aging: insights from reversibly immortalized keratinocytes

**DOI:** 10.18632/aging.206117

**Published:** 2024-09-23

**Authors:** Lu-Wen Xu, Yi-Dan Sun, Qiao-Yu Fu, Dan Wu, Jian Lin, Chen Wang, Liang Zhang, Cai-Yue Liu, Qing-Feng Li

**Affiliations:** 1 Department of Plastic and Reconstructive Surgery, Shanghai Ninth People’s Hospital, Shanghai Jiao Tong University School of Medicine, Shanghai 200021, China; 2 Department of Plastic and Reconstructive Surgery, Changzheng Hospital, Naval Medical University, Shanghai 200003, China; 3 Department of Center for Orthopedic Repair and Reconstruction, Chongming Hospital Affiliated to Shanghai University of Medicine and Health Sciences, Shanghai 202150, China; 4 CAS Key Laboratory of Tissue Microenvironment and Tumor, Shanghai Institute of Nutrition and Health, Chinese Academy of Sciences, University of Chinese Academy of Sciences, Shanghai 200031 China; 5 Shanghai Key Laboratory of Reproductive Medicine, Shanghai, China

**Keywords:** keratinocytes, epidermis, aging, immortalized cells, SV40T

## Abstract

Aging of epidermal keratinocytes profoundly impacts skin health, contributing to changes in appearance, barrier function, and susceptibility to diseases. Despite its significance, the molecular mechanisms underlying epidermal aging remain elusive. In this study, a reversible immortalized cell line was established by expressing SV40T in keratinocytes using the Tet-Off lentiviral system. Inducing a senescent phenotype by terminating SV40T expression revealed a significant reduction in mitotic ability, as well as characteristics of cellular aging. RNA sequencing analysis revealed alterations in gene expression and signaling pathways including DNA repair dysfunction, notably senescence-associated secretory phenotype (SASP)-related genes, such as MMP1, SERPINB2 and VEGFA. Our study provides insights into the molecular mechanisms of epidermal aging, offering potential therapeutic targets and highlighting the role of SASP in the aging process.

## INTRODUCTION

Aging, or senescence, can be defined as the gradual decline in the physiological functions and integrity of an organism, typically associated with increasing chronological age [1]. Aging of epidermal keratinocytes, the predominant cell type in the human epidermis, is a complex biological process that influences not only individual cell fate but also the overall physiology of the skin. The aging of keratinocytes has profound implications for skin health, contributing to changes in appearance, barrier function, ability of wound healing, and susceptibility to various skin diseases [2]. During aging, keratinocytes undergo significant changes including alterations in their proliferative capacity, differentiation, and response to environmental stimuli [3]. However, the underlying mechanism of epidermal aging remains largely elusive.

A growing body of literature has focused on understanding the molecular and cellular mechanisms underlying keratinocyte senescence. However, the exploration of the senescence-associated secretory phenotype (SASP) in human epidermal keratinocytes remains a relatively uncharted area. The SASP is defined as a complex and dynamic set of secretory activities exhibited by senescent cells, which is considered a key feature of cellular senescence and is closely associated with aging and age-related diseases [4]. It was reported that a mouse model with induced mitochondrial dysfunction led to an atypical form of SASP [5], which lacked the significant inflammatory components usually seen in senescence of human and mouse keratinocytes, and also resulted in accelerated keratinocyte differentiation. Despite this, the mice displayed characteristics of skin aging, and the findings also support the idea that the differentiation process might be the method for the removal of aging keratinocytes. Although there is limited definition and research on the SASP in human keratinocytes, the existing studies suggest that SASP potentially plays a significant role in the aging of the human epidermis.

In this study, we established a reversibly immortalized cell line by expressing SV40T in keratinocytes using the Tet-Off lentiviral system. Subsequently, we induced a senescent phenotype by terminating the expression of SV40T with doxycycline induction. SV40T antigen is known for its role in cell cycle regulation by inactivating key tumor suppressor proteins such as p53 and the retinoblastoma protein (Rb), which allows the cells to bypass normal senescence checkpoints and continue dividing. It was found that subsequently knocking out the introduced SV40T gene in immortalized epithelial cells leads to telomere shortening, ultimately leading to a state of premature senescence [6]. We verified that the approach of terminating the expression of SV40T gene led to keratinocyte senescence, providing a unique model to study keratinocyte aging. Subsequent RNA sequencing aims to uncover changes in gene expression and related signaling pathways, with a particular focus on SASP-related genes. Our research offers the potential to deepen our understanding of the molecular mechanisms of keratinocyte senescence and its impact on skin aging.

## RESULTS

### Reversing the SV40T expression in reversibly immortalized NHEK resulted in reduced mitotic ability

**Figure 1:**
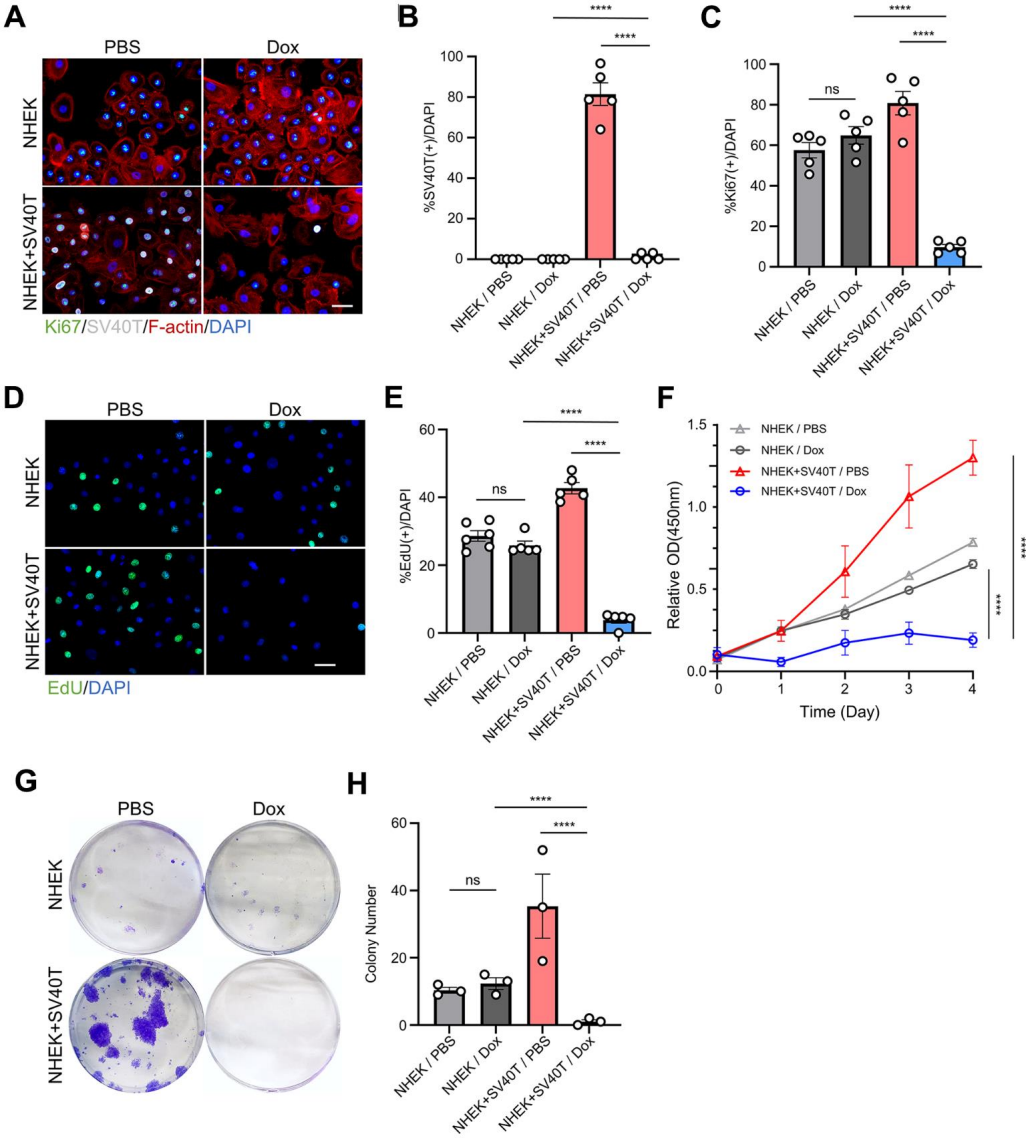
**Terminating the expression of SV40T antigen with doxycycline induction in reversibly immortalized NHEK resulted in reduced mitotic ability.** (**A**) Immunofluorescent staining of Ki67, SV40-T, F-actin and DAPI of NHEKs and reversibly immortalized keratinocytes with PBS or Dox treatment. Scale bar = 20 μm. (**B**, **C**) Statistical analysis for (**A**) (****P < 0.0001). (**D**) EdU staining analysis of NHEKs and reversibly immortalized keratinocytes with PBS or Dox treatment. Scale bar = 20 μm (****P < 0.0001). (**E**) Statistical analysis for (**D**) (****P < 0.0001, ns indicates not significant). All data are represented as mean +/- SEM. (**F**) CCK-8 assay for NHEKs and reversibly immortalized keratinocytes treated with either doxycycline (Dox) or PBS control (****P < 0.0001). (**G**) Clone formation assay for NHEKs and reversibly immortalized keratinocytes treated with either Dox or PBS control. (**H**) Statistical analysis for (**G**) (****P < 0.0001, ns indicates not significant).

To investigate the effects of the expression and subsequent terminalization of immortalization genes on the human keratinocytes, we established reversibly immortalized NHEK utilizing lentiviral vector containing the SV40T gene with doxycycline-inducible Tet-Off promoter. The transfection of the Tet-Off system allows the continuous expression of the target gene, in our case, SV40T antigen, and subsequently stops its transcription once the tetracycline or doxycycline were added to the cell culture. We initially assessed the expression of SV40T in Tet-Off-SV40T-transfected NHEKs and in sham NHEKs with immunofluorescence staining. In normal keratinocytes, there was no detectable expression of SV40T antigen in neither PBS nor Dox group. However, in cells transfected with the constructed Tet-Off-SV40T plasmid, SV40T antigen was expressed in over 80% of the cells. Furthermore, the addition of doxycycline (Dox) almost completely abolished the expression of SV40T in these cells, confirming the effectiveness of our Tet-Off system and the successful establishment of the reversibly immortalized keratinocytes (Figure 1A, 1B). Immunofluorescence staining also revealed that the expression of Ki67 in immortalized cells is significantly upregulated compared to normal cells, and with the doxycycline-induced suppression of SV40T expression, there presented a significant decrease in Ki67 expression, falling below the levels observed in normal cells (Figure 1A, 1C). EdU staining experiments demonstrated a similar trend (Figure 1D, 1E). CCK-8 assay was performed on both sham NHEKs and reversibly immortalized NHEKs with either PBS control or Dox. The CCK-8 curves showed an upregulated proliferation capacity in immortalized NHEKs, while subsequently terminating the SV40T genes resulted in a vast drop in the curve (Figure 1F). In the clonogenic assay, it can be observed that doxycycline did not significantly affect the clonogenic ability of sham NHEKs. However, in reversible immortalized cells, the number of cell colonies formed under doxycycline induction is significantly reduced, contrasting with the significant number of clone clusters in the immortalized cell group (Figure 1G, 1H). The aforementioned findings indicate that, subsequent to the inactivation of the SV40T gene, reversibly immortalized cells do not revert to their initial proliferative state. Rather, cellular proliferation decreases drastically, with little to non-colony forming abilities.

### Reversed immortalized NHEK showed phenotypes of cell senescence

**Figure 2:**
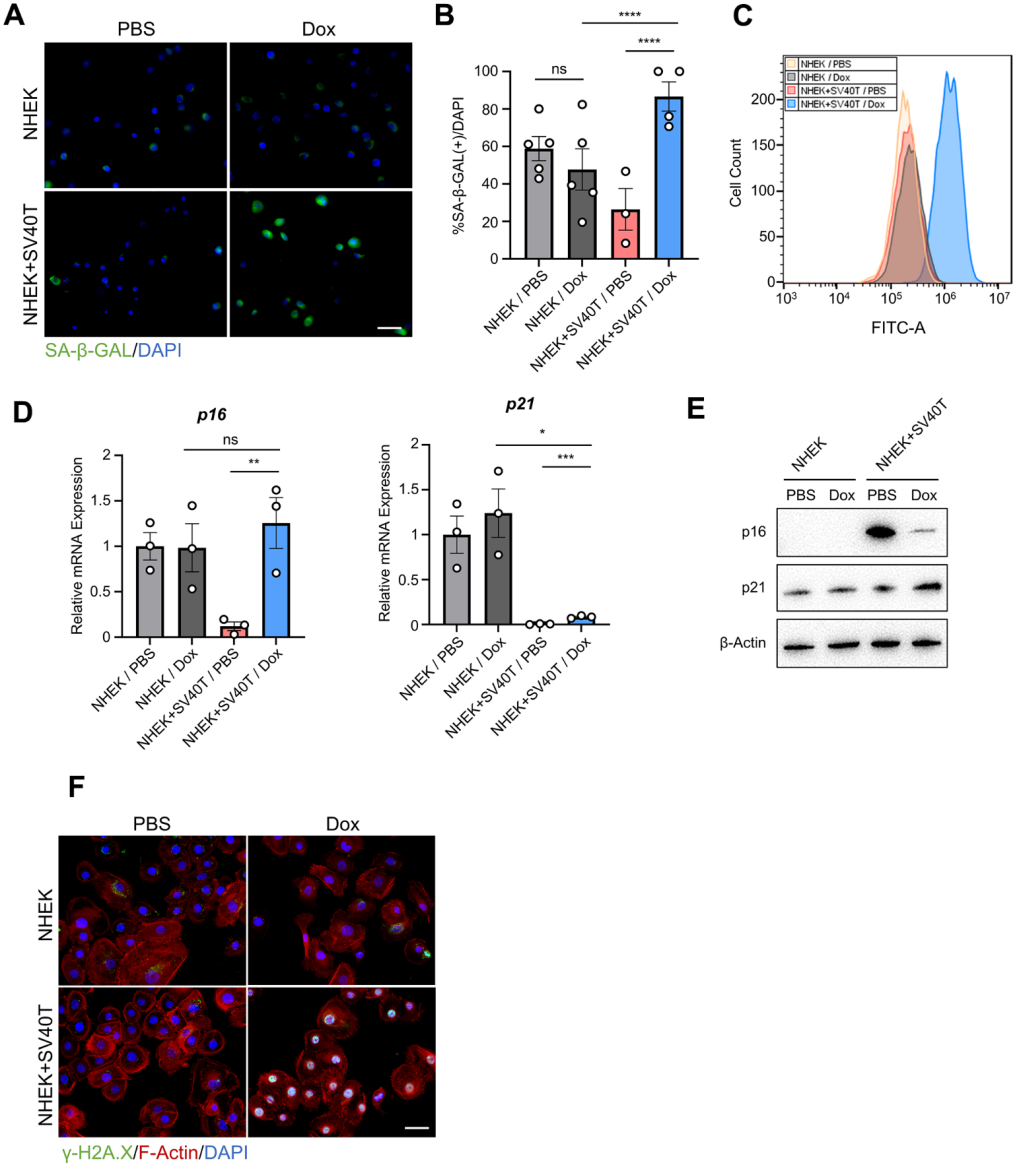
**Immortalized NHEK with terminated expression of SV40T showed phenotypes of cell senescence.** (**A**) Immunofluorescence detection of SA-β-GAL in NHEKs and reversibly immortalized keratinocytes with PBS or Dox treatment. Scale bar = 20 μm. (**B**) Statistical analysis of (**B**) (****P < 0.0001, ns indicates not significant). (**C**) Flow cytometry analysis of SA-β-GAL in NHEKs and reversibly immortalized keratinocytes with PBS or Dox treatment. (**D**) mRNA levels of *p16* and *p21* were tested by qPCR with corresponding treatment (*P < 0.05, **P < 0.01, ***P < 0.001, ****P < 0.0001, ns indicates not significant). (**E**) Protein levels of p16 and p21 were tested by Western blot in NHEKs and reversibly immortalized keratinocytes with corresponding treatment. (**F**) Immunofluorescent staining of γ-H2A.X, F-actin and DAPI of NHEKs and reversibly immortalized keratinocytes with PBS or Dox treatment. Scale bar = 20 μm.

To verify whether terminating the expression of SV40T resulted in cell senescence, we conducted the SA-β-GAL assay on both NHEK and reversibly immortalized keratinocytes. The immunofluorescence test suggested higher expression of SA-β-GAL in reversed immortalized cells compared to either NHEKs or immortalized cells (Figure 2A, 2B). Similar results were observed with Flow cytometry assays detecting SA-β-GAL, with a shift in the peak of reversed immortalized cells (Figure 2C). Analysis at the transcriptomic level revealed an upregulation of the senescence markers *p16* and *p21* (Figure 2D). However, the augmented expression of *p16* in senescent epidermis remains contentious. Previous study has posited that melanocytes were the sole cell type exhibiting elevated p16 expression in aged epidermis [7], while in other studies p16 continues to serve as a marker of senescence in keratinocytes under exogenous stress [8] [9]. Notably, our findings present inverse correlation between protein and mRNA levels (Figure 2E), which might validate that p16 may not be a suitable marker of aging in epidermal cells. Additionally, γ-H2A.X expression in the nuclei of reversed immortalized cells were upregulated, indicating an increase in DNA damage (Figure 2F). To conclude, reversed immortalized cells exhibit cell senescent phenotypes, characterized by the expression of canonical cell aging biomarkers and the accumulation of DNA damage.

### RNA-sequencing analysis revealed signaling pathways related to epidermal senescence

**Figure 3:**
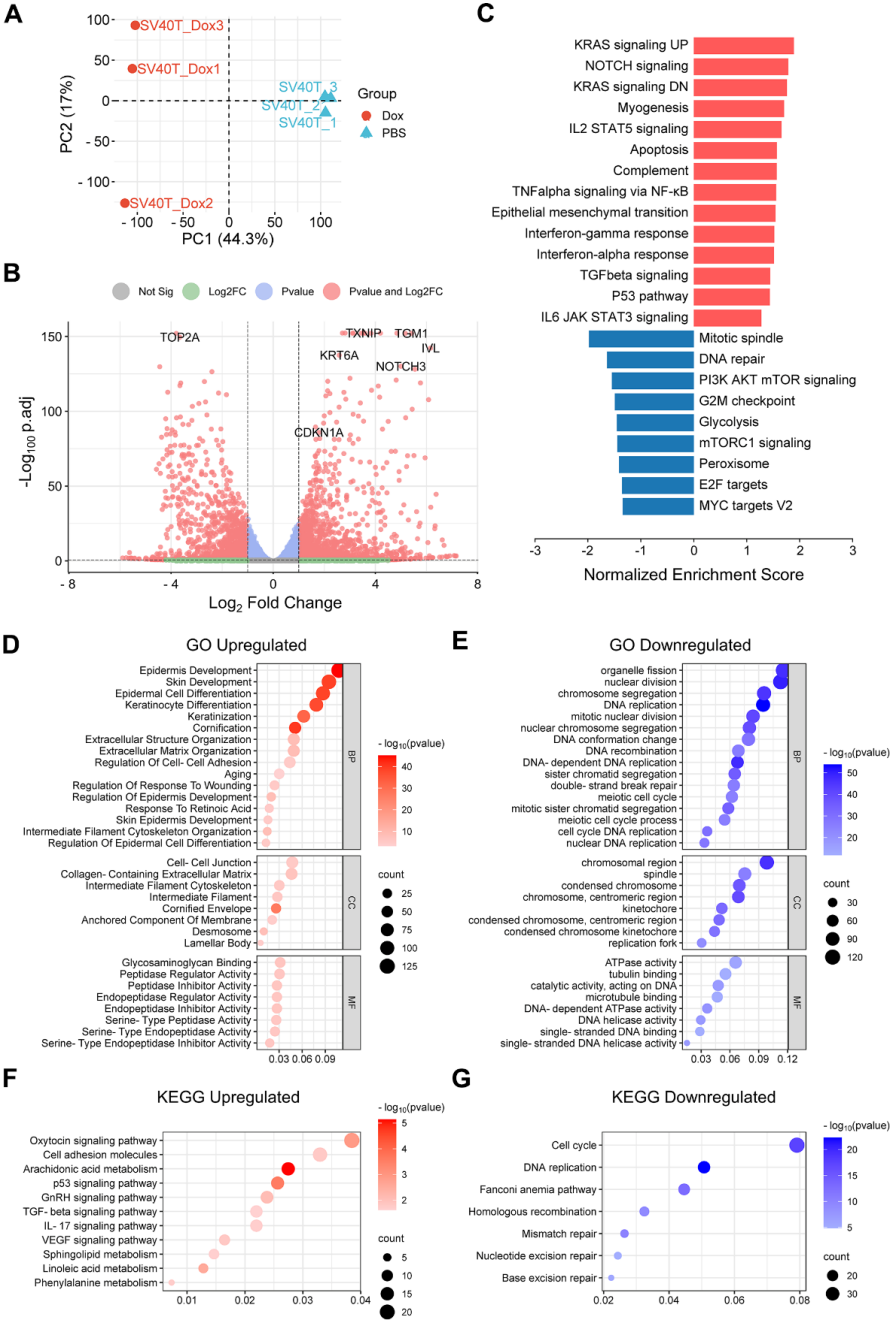
**Result of RNA-sequencing analysis of reversibly immortalized NHEK with Dox induction or PBS control.** (**A**) Principal component analysis (PCA) plot of gene expression of biological triplicates from RNA-seq of reversibly immortalized NHEK with PBS or Dox treatment. (**B**) Volcano plot of up- or downregulated genes in reversibly immortalized NHEK with PBS or Dox treatment with top-ranked genes annotated. (**C**) Results of Gene Sets Enrichment Analysis (GSEA) of the Hallmark gene sets for DEGs between PBS and Dox group, with red bars showing enriched terms in upregulated genes and blue bars showing enriched terms in downregulated genes. (**D**) Bubble plot of GO enrichment analysis of upregulated DEGs. (**E**) Bubble plot of KEGG pathway enrichment analysis of upregulated DEGs. (**F**) Bubble plot of GO enrichment analysis of downregulated DEGs. (**G**) Bubble plot of KEGG pathway enrichment analysis of downregulated DEGs.

To further investigate the potential molecular mechanisms underlying the senescence phenotype of reversed immortalized keratinocytes, we performed RNA sequencing on Dox-induced senescent cells and PBS control cells. PCA was used to distinguish the overall distribution trend between two groups of samples (Figure 3A). There is no crossover between the Dox group and the PBS group, which shows that the difference between the two groups is relatively large, indicating that the transcriptome between the immortalized keratinocytes and SV40T-reversed senescent group tend to separate. 2962 differentially-expressed genes between Dox group and PBS group were identified, including 1631 upregulated genes and 1331 downregulated genes in the context of Dox group. Overall distribution of DEGs was presented in the volcano plot with the annotation of top-ranked DEGs (Figure 3B).

To explore the biological function of DEGs, GSEA analysis was performed to identify the potential pathways related to senescent phenotypes of the Dox group. Significant enriched signaling pathways from Hallmark database were chosen based on both adjusted P-value (<0.05) and FDR q-value (<0.25). Typical enriched signaling pathways from were shown in Figure 3C, with bars representing upregulated pathways colored as red and those representing down regulated pathways colored as blue. KRAS signaling pathway was activated along with Notch signaling, myogenesis, IL2-STAT5 signaling, apoptosis and NF-κB signaling etc. Thereinto, Notch signaling pathway, TGFβ signaling via NF-κB and IL6/JAK/STAT3 signaling pathway were known to be related to cell senescent [10–12]. In contrast, signaling pathways related to cell cycle and division were downregulated, represented by mitotic spindle, mTOR signaling and G2M checkpoint.

GO and KEGG pathway analysis were also conducted for further investigation. Under the condition of p-value<0.05, upregulated DEGs were involved in 1072 biological process (GO-BP), 62 cell component (GO-CC), 122 molecular function (GO-MF) and 34 KEGG, while downregulated DEGs were involved in 750 biological process (GO-BP), 94 cell component (GO-CC), 105 molecular function (GO-MF) and 30 KEGG. The bubble graph demonstrates typical messages for GO-BP, GO-CC, GO-MF and KEGG, respectively (Figure 3D–3G). GO functional annotations showed that upregulated DEGs were mainly involved in epidermis development, epidermis differentiation, extracellular matrix organization and aging. Meanwhile, downregulated DEGs were mainly involved in cell metabolism, mitotic activity and DNA repair. KEGG pathway analysis demonstrated that the DEGs were primarily associated with upregulated cell adhesion, arachidonic acid metabolism and p53 signaling pathway, as well as downregulated cell cycle and DNA replication.

### Reversed immortalized keratinocytes showed enhancement of SASP

**Figure 4:**
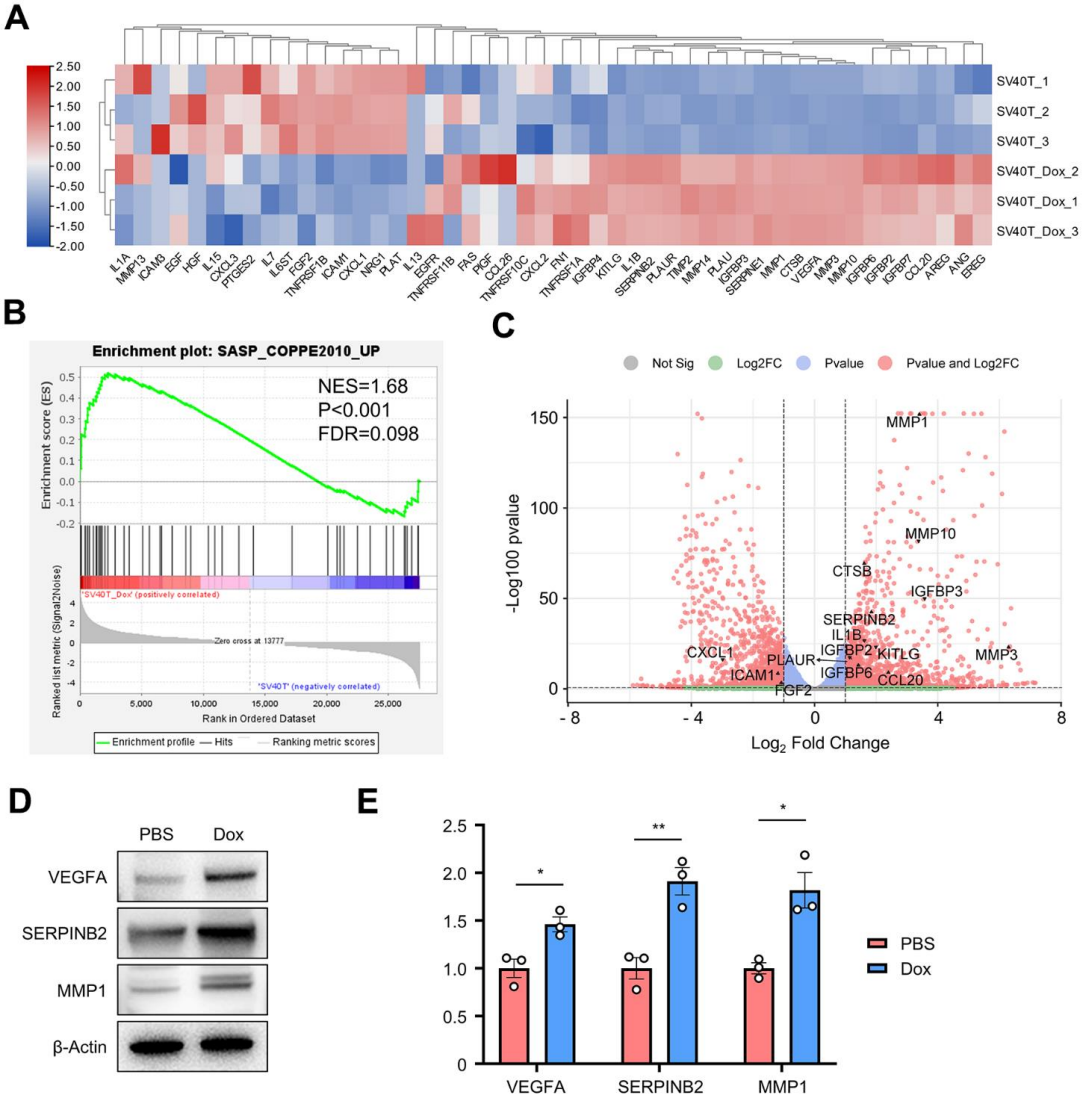
**Gene set enrichment analysis revealed enrichment of SASP-related genes set in the DEGs.** (**A**) Gene expression heatmap of SASP-related genes in reversibly immortalized NHEK with PBS or Dox treatment. (**B**) GSEA results of SASP gene set. (**C**) Volcano plot of up- or downregulated genes in reversibly immortalized NHEK with PBS or Dox treatment with significant genes in SASP gene set annotated. (**D**) Protein levels of VEGFA, SERPINB2 and MMP1 were tested by Western blot in reversibly immortalized keratinocytes with PBS or Dox treatment. (**E**) mRNA levels of VEGFA, SERPINB2 and MMP1 were tested by qPCR in reversibly immortalized keratinocytes with PBS or Dox treatment.

Cellular senescence leads to an irreversible arrest of the cell cycle and induces significant phenotypic alterations, including the generation of a series of bioactive secretome, known as the senescence-associated secretory phenotype (SASP). To identify whether SASP plays a key role in the senescence of reversed immortalized keratinocytes, a GSEA gene set for senescence-associated genes was acquired from previous studies (Supplementary Table 1) [13, 14]. 48 genes within the SASP-related gene set were found expressed in our cell samples, with their expression plotted in the clustered heatmap (Figure 4A). More than half of those genes were generally upregulated in Dox group, including MMPs, IGFBPs, SERPINs, interleukins and growth factors. GSEA analysis resulted in significant enrichment in such SASP-related gene set with NES=1.68, p-value<0.001 and FDR=0.098 (Figure 4B). Top significant genes were annotated in the volcano plot of all DEGs (Figure 4C). MMP3, SERPINB2 and VEGFA as typically expressed proteins from SASP results were verified upregulated in reversed immortalized keratinocytes in both Western blot and qPCR (Figure 4D, 4E).

### Senescent organotypic 3D skin models constructed with reversed immortalized keratinocytes presented enhanced feature of SASP

**Figure 5:**
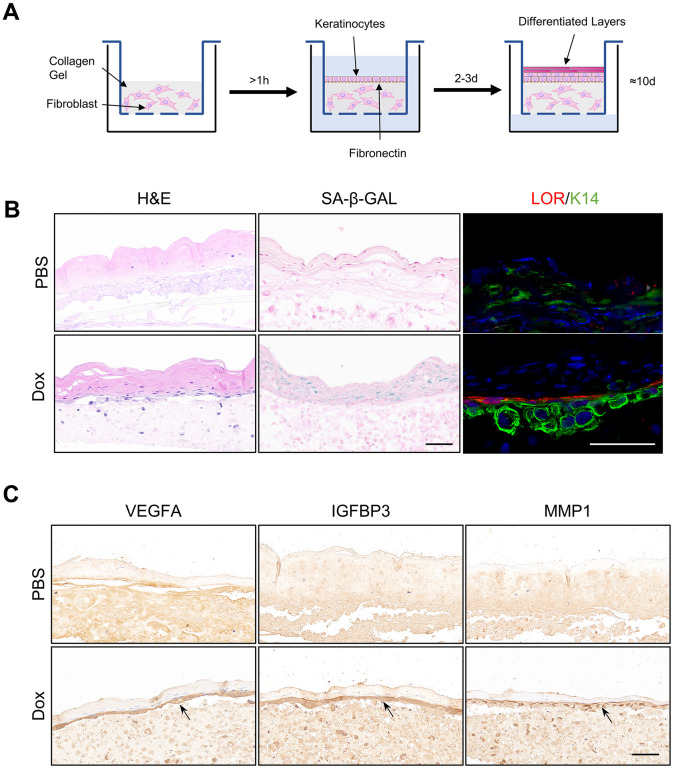
**Organotypic 3D skin model constructed with reversibly immortalized NHEK showed aging phenotypes with Dox induction.** (**A**) Flowchart of organotypic skin culture. (**B**) H&E staining, SA-β-GAL staining, and immunofluorescent staining of Loricrin and K14 in organotypic skin cultures constructed with reversibly immortalized keratinocytes with PBS or Dox treatment. Scale bar = 20 μm. (**C**) IHC staining analysis of SASP-related proteins VEGFA, IGFBP3 and MMP1 in organotypic 3D skin cultures constructed with reversibly immortalized keratinocytes with either PBS or Dox treatment. Scale bar = 20 μm.

To further verify above results, full-thickness organotypic 3D skin models were constructed with reversibly immortalized keratinocytes as described (Figure 5A, Methods and Materials). Structure of normal epidermis, including basal layer, differentiated layer and cornified envelope were observed in the H&E staining of Organotypic 3D skin models. Staining of SA-β-GAL in the epidermis was upregulated in the ones composed of reversed immortalized keratinocytes with Dox induction for more than one week, indicating the aging of organotypic epidermis. Immunofluorescence staining of Loricrin exhibited enhanced differentiation, consistent with previous results from RNA-sequencing (Figure 5C). However, we noted that the marker of epidermal basal cells, K14, is downregulated in immortalized cells within the 3D model, which may imply the loss of stemness, of which the reason remains to be debated. Finally, we utilized such senescent organotypic 3D skin model to validate the level of the SASP. Our findings indicate a notable increase in the expression of representative SASP proteins VEGFA, IGFBP3, and MMP1 in the epidermis of the senescent 3D skin models treated with Dox, particularly in the basal layer. These results suggest that the increase of SASP is predominantly localized to the basal cells of the epidermis, which may play a crucial role in driving the aging process of the epidermis. The location of the increase in SASP factors may also imply the contribution of basal keratinocytes to the structural and functional alterations observed in aged epidermal tissue.

### Senescent reversed immortalized keratinocytes presented different genetic characteristic with replicative senescent fibroblasts

**Figure 6:**
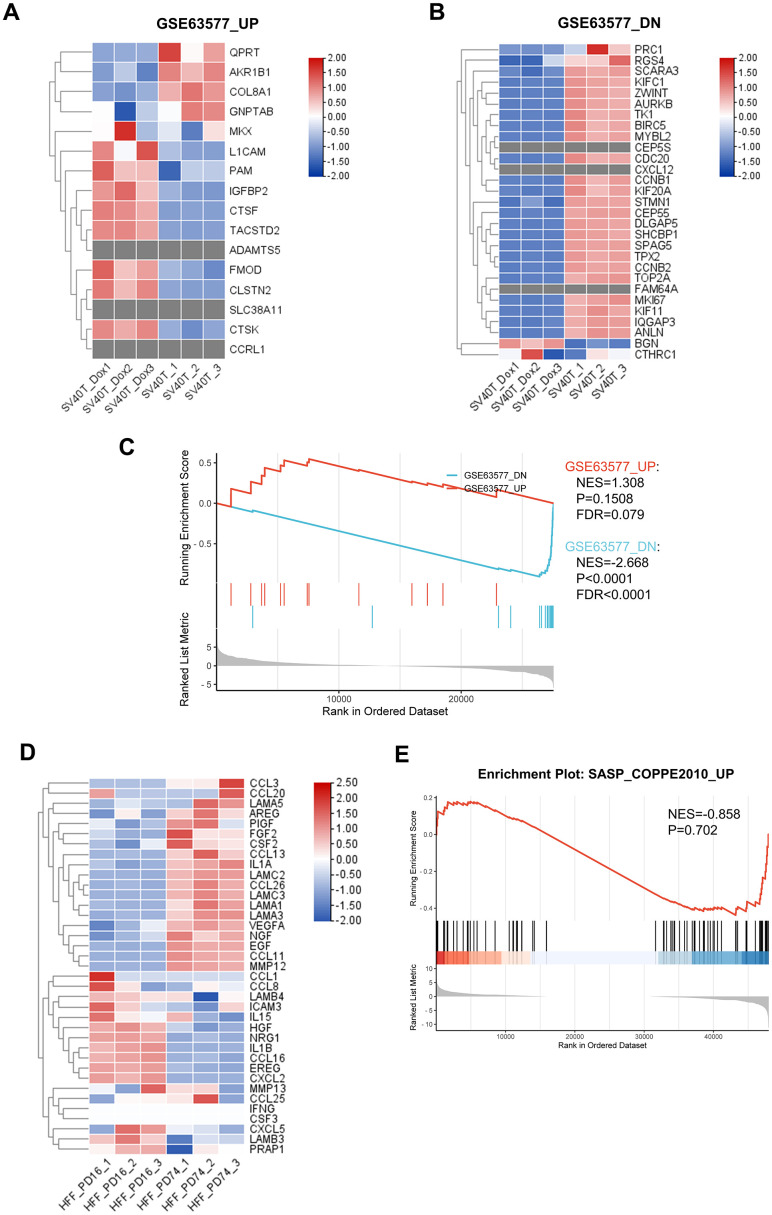
**Analysis of RNA sequencing data demonstrated distinct gene expression pattern between replicative senescent dermal fibroblasts and senescent reversed immortalized keratinocytes.** (**A**, **B**) Gene expression heatmaps illustrating the expression profiles of gene sets previously identified as upregulated and downregulated in senescent dermal fibroblasts, according to data from GSE63577. (**C**) GSEA results depicting the enrichment of DEGs between PBS and Dox group of reversibly immortalized keratinocytes in gene sets from GSE63577 dataset. (**D**) Gene expression heatmap showing the expression of the SASP-related gene set in senescent fibroblasts from the GSE63577 dataset. (**E**) GSEA result of the enrichment of DEGs from the GSE63577 dataset in the SASP-related gene set.

While a significant proportion of research into skin aging has focused on dermis and dermal fibroblasts, emerging strategies suggest a critical examination of epidermal components is equally pivotal. To this end, we employed reversibly immortalized keratinocytes as a model to study epidermal aging, providing a comparative analysis with replicative senescent fibroblasts, in order to uncover the distinct genomic signatures between the epidermal and dermal aging processes. We revised the RNA sequencing data from published research on replicative senescent dermal fibroblasts [15] and identified key gene sets that were notably upregulated or downregulated. Corresponding gene expression patterns in our keratinocyte model was assessed and presented in the heatmaps (Figure 6A, 6B). Interestingly, the analysis revealed discrepancies in gene expression; several genes upregulated in senescent fibroblasts were found to be downregulated in keratinocytes, and vice versa. Furthermore, certain genes observed in fibroblasts were completely absent in the keratinocytes. To confirm our insights, we conducted GSEA on both our RNA-seq data and the published fibroblast datasets (Figure 6C). The results indicated a failure of enrichment for gene sets that were upregulated in the aging fibroblasts within our keratinocyte model, suggesting divergent aging mechanisms between these cell types. Additionally, our GSEA of the DEGs of senescent fibroblasts in SASP-related gene set came back negative, therefore indicated that SASP in aging skin might be predominantly epidermal-specific.

## DISCUSSION

SV40 large T antigen, a protein derived from the polyomavirus SV40, is widely used for the establishment of immortalized cell lines [16]. SV40T antigen binds and inactivates tumor suppressor proteins p53, causing the cells to leave G1 phase and entering into S phase, which promotes DNA replication [17]. Previous research has demonstrated that human foreskin epidermal cells immortalized by expressing SV40T antigen exhibited telomere shortening and premature senescence following the subsequent excision of SV40T with Cre-loxP system [6]. However, in these studies, only the characteristics of replicative senescence, namely telomere shortening, were identified, without further investigation into the underlying molecular mechanisms.

In this study, we utilized the Tet-Off lentiviral system to establish a reversible immortalized cell line in which SV40T expression could be terminated upon doxycycline induction. We discovered that once the expression of SV40T was terminated, the proliferative capacity of these cells not only reverted to the level before immortalization but was even significantly lower than that of normal cells. Investigation of aging markers revealed that these cells were in a state of premature senescence. Notably, previous studies have reported that the upregulation of p16 expression in aging epidermis remains a contentious topic. According to the research by Victorelli et al. [7], melanocytes were the only cell type in aging epidermis that upregulates p16 expression, excluding keratinocytes. Our results, showing inconsistent expression patterns in p16 expression at the transcript and protein levels, also lend support to this perspective. Nevertheless, we observed an upregulation in the expression levels of p21 and SA-β-galactosidase, along with the expression of γ-H2AX in the nucleus. These findings indicate that the senescence of reversed immortalized keratinocytes was accompanied by DNA damage. Next, we further investigated the senescent phenotypes and its underlying mechanisms of reversed immortalized keratinocytes with bulk RNA sequencing technology. Analysis of DEGs between reversed and non-reversed immortalized cells indicated enrichment of pathways involved in keratinocyte aging, including Notch signaling pathway and TNFα signaling pathway via NF-κB, as well as impaired DNA repair and cell cycle.

However, we observed an enhancement in the differentiation capacity of the reversed immortalized keratinocytes. Conventionally, it is believed that epidermal aging leads to a diminution in the differentiation ability of keratinocytes, consequently thinning the stratum corneum of the skin. Additionally, studies have shown that the suppression of p53 in human keratinocytes induces squamous differentiation [18]. Theoretically, the cessation of SV40T’s inhibition on p53 should result in a downregulation of cellular differentiation, contrary to the upregulated pattern observed in our conclusions. To explain this outcome, we hypothesize that the continuous induction of cells into the cell cycle by SV40T disrupts the potential differentiation pathways, which were restored by the termination of SV40T expression. Concurrently, the termination of cell replication via p21-induced mitotic block may lead to replicative stress, resulting in squamous differentiation [19]. Furthermore, previous studies have also identified that mitochondrial dysfunction in murine keratinocytes leads to atypical SASP and AMPK-mediated p53 activation accompanied by accelerated cell differentiation [5], a finding that parallels our research results. Combining multiple hypotheses, we believe that this phenomenon is not a mere upregulation of differentiation, calling for further investigation. Given the aforementioned results, we posit that reversed immortalized keratinocytes may serve as an interesting *in vitro* model for epidermal aging. This model, in comparison to traditional replicative senescence models, demonstrates higher construction efficiency and offers easier acquisition and cultivation than keratinocytes derived from skin samples of elderly patients.

The SASP is a phenotype characterizing senescent cells that exhibit heightened secretion of various substances, including inflammatory cytokines, immune modulators, growth factors, and proteases [20, 21]. Many current studies have shown the role of SASP in skin aging, highlighting factors including IL-6, IL-8, IFN-γ and such [14]. To this day, the majority of research on the SASP within human skin has been centered on dermal fibroblasts, with limited understanding of the SASP in other skin-resident cell types such as keratinocytes [22]. In our cellular model, we detected an enrichment of the SASP, including various cytokines and proteases such as MMP1, MMP3, IGFBP3, IL-1β, SERPINB2, and VEGFA, which varies from those identified in dermal fibroblasts [23]. Specifically, genes such as IL1B, which was identified in our study as upregulated, play crucial roles in mediating inflammatory responses, and was also recognized as a crucial feature of “inflammaging” [24]. Inflammaging refers to the phenomenon of chronic inflammation within the cells and tissues during aging, which is also characterized by elevated level of metalloproteinases. The enhanced expression of these genes in senescent cells not only supports the persistence of a pro-inflammatory environment but also underlines the potential of SASP factors to drive aging-related pathologies. The activation of NF-κB, as identified in our enrichment analysis, was reported to serve as a driving factor of the SASP [25]. Senescent cells were also found to communicate with their microenvironment through juxtacrine NOTCH signaling [26]. We posit that in this study, the exocrine of SASP-related factors under the restored activation of p53 contribute to the inability of the reversed immortalized cells to maintain their original proliferation levels, leading to the accumulation of senescence. Therefore, our research provides potential molecular hallmarks for the characterization of SASP in the epidermis.

Finally, our study elucidates distinct molecular phenotypes between aging epidermal keratinocytes and dermal fibroblasts, including discrepancies in gene expression pattern and predominant presence of SASP in epidermal cells, suggesting that unique molecular mechanisms drive aging in different skin cell types. These results underscore the need for targeted aging interventions tailored to specific cellular environments within the skin.

In conclusion, the study opens new avenues for understanding the molecular mechanisms of epidermal aging. Yet, the characteristics of the SASP in the human epidermis warrant further investigation and validation. Crucial aspects that remain to be explored include identifying specific pathways that play a pivotal role in the aging of epidermal cells, strategies for intervening in and potentially reversing cellular senescence, and evaluating the effectiveness of existing anti-aging drugs on epidermal aging. Additionally, the interaction of SASP between different cell types in the epidermis and keratinocytes, and the distinctions between replicative senescence and oxidative stress-induced aging, present further intriguing areas of research. This study thereby not only sheds light on the complex aging process in epidermal cells but also sets the stage for future explorations into therapeutic interventions finely tailored to the unique characteristics of epidermal aging and a deeper understanding of skin aging at the cellular level.

## MATERIALS AND METHODS

### Cell culture

Normal Human Epidermal Keratinocytes (NHEK) and Normal Human Dermal Fibroblasts (NHDF) were obtained from juvenile human foreskin samples from plastic surgery wastes with informed patient consent. Briefly, skin biopsies were washed once in 70% ethanol and twice in PBS to sterilize and wash out the remaining blood. Scrape off the hypodermis with a scalpel blade and cut the skin sample into smaller fragments. The samples were then digested overnight in 2mg/ml Dispase II (Roche, Switzerland) solution at 4° C. Next day, peel off the epidermis from the dermis using the forceps and mince them separately. The epidermis was treated with Trypsin-Versene (Lonza, Switzerland) for 15 min at 37° C before the NHEKs were collected and cultured in keratinocyte medium (CELLnTEC Switzerland). The dermis was treated with 2.5 mg/mL collagenase for 45 min at 37° C before the NHDFs were collected and cultured in complete DMEM medium. Cells were incubated at 37° C in a 95% humidified atmosphere and 5% CO_2_. The culture medium was changed every 2–3 days, and cells were passaged when confluency reached approximately 80–90%. Only early passages of keratinocytes (passage 3–6) and fibroblasts (passage 3–10) were used in this study.

### Reversible expression of SV40T antigen in NHEKs

Reversible SV40T antigen expression in NHEKs was achieved by lentivirus infection. HEK293T cells were cultivated in DMEM supplemented with 10% FBS and 1% Pen/Strep (Thermo Fisher Scientific, USA). The lentivirus and negative controls were prepared by mixing pMD2-VSVG, pPAX2, and the lentiviral plasmid (either pLVX-TetOff-SV40-T-BsdS or corresponding vector control) in serum-free medium and PEI solution (w/v=1:3), and adding into HEK293T with 90% confluence. After 16h of incubation, the medium was changed to normal complete medium. Supernatant containing lentivirus was collected at both 48h and 72h, filtrated through 0.45 μm filter. The NHEKs in the logarithmic growth were plated onto the 6-well culture plate. When 50-60% confluence was reached, add certain amount of virus and 100 μg/mL polybrene into the 1mL medium. The infected NHEKs were incubated overnight before changing into the normal medium. 100 μg/mL Blasticidin S was added to the medium from Day 2 for 4 days. 4 μg/mL doxycycline was used to induce Tet-Off promoter.

### Cell proliferation (CCK-8) assay

Cell proliferation assay was delivered according to manufacturer’s instruction (Beyotime, China). Cells were plated into 96-well plates for 3000 cells per well. Measurement was made every day after incubation with 10 μL of CCK-8 solution for 2h, by absorbance reading using a microplate reader at a wavelength of 450 nm.

### Cloning formation assay

Cells in the logarithmic growth phase were seeded onto the 6-well plate for 500 cells per well. After 2 weeks of culture, the cells were fixed with 4% paraformaldehyde and stained with crystal violet. The stained cells were then observed, photographed, and counted with ImageJ.

### Histological and immunofluorescence analysis

Organotypic cultures were harvested and cut into strips with scalpel blades before being immediately embedded in OCT compound and frozen at -20° C for immunofluorescence analysis, or fixed in 4% paraformaldehyde and embedded in paraffin for section and H&E staining.

For cell immunofluorescence analysis, keratinocytes were plated at 1000/wells onto glass coverslips and grown as previously described. Cells on coverslips or frozen section of organotypic cultures were fixed in 4% paraformaldehyde for 10 min at room temperature, carefully washed PBS and blocked in Blocking Solution (2.5% Normal Goat Serum, 2.5% Normal Donkey Serum, 1% BSA, 0.3% Triton-X 100 in PBS) for 1h at room temperature. Primary antibodies diluted in Blocking solution were incubated at 4° C overnight. Secondary antibodies diluted in Blocking Solution were incubated for 1h at room temperature. Mounting medium with DAPI was used to mount coverslips. Zeiss Axio Imager A2 (Zeiss, Germany) was used to visualize fluorescent signals.

### SA-β-GAL detection

Detection of SA-β-GAL in keratinocytes was performed utilizing commercial kits from Dojindo Molecular Technologies, Inc. (SG003, Japan) according to the given instruction. Detection of fluorescence was conducted by both microscopic observation method and the flow cytometry method. Detection of SA-β-GAL in frozen sections of organotypic 3D skin models was performed using SA-β-Gal Stain Kit from Solarbio Science & Technology Co. (G1580, China).

### qRT-PCR analysis

Total RNA was extracted from either keratinocytes or organotypic skin cultures using RNA Easy Fast Tissue/Cell Kit (Tiangen, China), which was then used to prepare cDNA utilizing the FastKing gDNA Dispelling RT SuperMix (Tiagen, China). After mixing cDNA with corresponding primers, the qRT-PCR reaction was run with SYBR Green reagent (Tiagen, China). All samples were analyzed based on 2^-ΔΔCT^ value method. GAPDH was utilized as an internal reference.

### Western blot

Protein level of p16 (18796, CST, USA), p21 (2947, CST, USA), MMP1 (10371-2-AP, Proteintech, USA), SERPINB2 (16035-1-AP, Proteintech, USA), VEGFA (66828-1-Ig, Proteintech, USA) in relative cells were analyzed by Western blotting using commercially available antibodies. The cell lysates were subjected to SDS-PAGE and subsequently transferred to a PVDF membrane (Epizyme, China). Following washes with TBST, protein bands were detected using enhanced chemiluminescence ECL (Epizyme, China) according to the manufacturer’s instructions.

### RNA sequencing and enrichment analysis

Total RNA was extracted using Trizol reagent (Thermo Fisher Scientific, USA). Library construction and sequencing were performed on the Illumina HiSeq 2000 platform at Novogene Inc., China, as well as with differential gene expression analysis. PCA analysis was conducted using FactoMineR R package (v2.9). Volcano plots were generated with EnhancedVolcano R package (v1.20.0).

Gene Set Enrichment analysis was performed using GSEA software (v4.3.2) on gene set collections H (Hallmark gene sets) downloaded from Molecular Signature Database (MSigDB) v7.0 with default settings. The gene set with an p.adj<0.05 and FDR<0.25 was considered as significantly enriched.

The gene ontology (GO) and Kyoto Encyclopedia of Genes and Genomes (KEGG) pathway analyses were performed using clusterProfiler R package (v4.10.0). Terms with p-value<0.05 were regarded as significantly enriched. The bubble plots were generated using ggplot2 R package (v3.4.4).

SASP-related gene set was derived from Coppé et al. [13]. RNA sequencing data of young and senescent human dermal fibroblasts along with its DEG sets were derived from Marthandan et al. [15] and its dataset GSE63577.

### Organotypic 3D skin model

Organotypic 3D skin model was constructed according to previous description with minor modification. The NHDFs were reconstituted in a collagen gel consisting of 80% 3mg/ml rat tail collagen (Thermo Fisher Scientific, USA), 10% 10× PBS, 10% FBS and 0.025mM NaOH at a density of 4 × 10^6^ cells/mL. The pH of fibroblast/collagen mixture was adjusted with 1mM NaOH to 7.0. Then, 400 μL of the fibroblast/gel mixture was added to each 0.4 μm pore insert in a 12-well plate (Biofil, China) and incubated at 37° C for 1h to allow for polymerization. 25 μl of 50 μg/ml fibronectin was incubated for 30min before 4 × 10^5^ NHEKs suspended in 0.5 mL of keratinocyte medium (8 × 10^5^ cells/mL) were added onto each fibroblast/collagen gel bed. 2 mL of keratinocyte medium was added to the outside of the inserts to reach the same liquid level as the inside of the inserts. The cells were incubated at 37° C for 2-3 days. After the cells were submerged, the insert was lifted to air-liquid interface by aspirating all the medium from the inside of the inserts and add Epilife Differentiation Medium to the outside that would just reach the insert membrane. Epilife Differentiation Medium was made by adding 1.15mM CaCl_2_, 50 μg/ml L-ascorbic acid, 0.1% bovine serum albumin, and 10 μg/ml transferrin to Epilife Medium. The skin culture was incubated at 37° C for another 10 days before harvest.

### Statistical analysis

Data are presented as means ± SEM with P-values calculated by two-tail student’s t-test. P < 0.05 was considered statistically significant.

## Supplementary Material

Supplementary Table 1

## References

[1] Höhn A, Weber D, Jung T, Ott C, Hugo M, Kochlik B, Kehm R, König J, Grune T, Castro JP. Happily (n)ever after: Aging in the context of oxidative stress, proteostasis loss and cellular senescence. Redox Biol. 2017; 11:482–501. 10.1016/j.redox.2016.12.001 28086196 PMC5228102

[2] Wang Z, Man MQ, Li T, Elias PM, Mauro TM. Aging-associated alterations in epidermal function and their clinical significance. Aging (Albany NY). 2020; 12:5551–65. 10.18632/aging.102946 32217811 PMC7138575

[3] Gruber F, Kremslehner C, Eckhart L, Tschachler E. Cell aging and cellular senescence in skin aging - Recent advances in fibroblast and keratinocyte biology. Exp Gerontol. 2020; 130:110780. 10.1016/j.exger.2019.110780 31794850

[4] Gorgoulis V, Adams PD, Alimonti A, Bennett DC, Bischof O, Bishop C, Campisi J, Collado M, Evangelou K, Ferbeyre G, Gil J, Hara E, Krizhanovsky V, et al. Cellular Senescence: Defining a Path Forward. Cell. 2019; 179:813–27. 10.1016/j.cell.2019.10.005 31675495

[5] Wiley CD, Velarde MC, Lecot P, Liu S, Sarnoski EA, Freund A, Shirakawa K, Lim HW, Davis SS, Ramanathan A, Gerencser AA, Verdin E, Campisi J. Mitochondrial Dysfunction Induces Senescence with a Distinct Secretory Phenotype. Cell Metab. 2016; 23:303–14. 10.1016/j.cmet.2015.11.011 26686024 PMC4749409

[6] Westerman KA, Leboulch P. Reversible immortalization of mammalian cells mediated by retroviral transfer and site-specific recombination. Proc Natl Acad Sci USA. 1996; 93:8971–6. 10.1073/pnas.93.17.8971 8799138 PMC38579

[7] Victorelli S, Lagnado A, Halim J, Moore W, Talbot D, Barrett K, Chapman J, Birch J, Ogrodnik M, Meves A, Pawlikowski JS, Jurk D, Adams PD, et al. Senescent human melanocytes drive skin ageing via paracrine telomere dysfunction. EMBO J. 2019; 38:e101982. 10.15252/embj.2019101982 31633821 PMC6885734

[8] Kang KA, Piao MJ, Fernando PDSM, Herath HMUL, Yi JM, Hyun JW. Korean Red Ginseng Attenuates Particulate Matter-Induced Senescence of Skin Keratinocytes. Antioxidants (Basel). 2023; 12:1516. 10.3390/antiox12081516 37627511 PMC10451201

[9] Miyake T, Shimada M, Matsumoto Y, Okino A. DNA Damage Response After Ionizing Radiation Exposure in Skin Keratinocytes Derived from Human-Induced Pluripotent Stem Cells. Int J Radiat Oncol Biol Phys. 2019; 105:193–205. 10.1016/j.ijrobp.2019.05.006 31085283

[10] Balistreri CR, Madonna R, Melino G, Caruso C. The emerging role of Notch pathway in ageing: Focus on the related mechanisms in age-related diseases. Ageing Res Rev. 2016; 29:50–65. 10.1016/j.arr.2016.06.004 27328278

[11] García-García VA, Alameda JP, Page A, Casanova ML. Role of NF-κB in Ageing and Age-Related Diseases: Lessons from Genetically Modified Mouse Models. Cells. 2021; 10:1906. 10.3390/cells10081906 34440675 PMC8394846

[12] Kojima H, Inoue T, Kunimoto H, Nakajima K. IL-6-STAT3 signaling and premature senescence. JAKSTAT. 2013; 2:e25763. 10.4161/jkst.25763 24416650 PMC3876432

[13] Coppé JP, Desprez PY, Krtolica A, Campisi J. The senescence-associated secretory phenotype: the dark side of tumor suppression. Annu Rev Pathol. 2010; 5:99–118. 10.1146/annurev-pathol-121808-102144 20078217 PMC4166495

[14] Waldera Lupa DM, Kalfalah F, Safferling K, Boukamp P, Poschmann G, Volpi E, Götz-Rösch C, Bernerd F, Haag L, Huebenthal U, Fritsche E, Boege F, Grabe N, et al. Characterization of Skin Aging-Associated Secreted Proteins (SAASP) Produced by Dermal Fibroblasts Isolated from Intrinsically Aged Human Skin. J Invest Dermatol. 2015; 135:1954–68. 10.1038/jid.2015.120 25815425

[15] Marthandan S, Baumgart M, Priebe S, Groth M, Schaer J, Kaether C, Guthke R, Cellerino A, Platzer M, Diekmann S, Hemmerich P. Conserved Senescence Associated Genes and Pathways in Primary Human Fibroblasts Detected by RNA-Seq. PLoS One. 2016; 11:e0154531. 10.1371/journal.pone.0154531 27140416 PMC4854426

[16] Fanning E. Simian virus 40 large T antigen: the puzzle, the pieces, and the emerging picture. J Virol. 1992; 66:1289–93. 10.1128/JVI.66.3.1289-1293.1992 1310750 PMC240849

[17] Ahuja D, Sáenz-Robles MT, Pipas JM. SV40 large T antigen targets multiple cellular pathways to elicit cellular transformation. Oncogene. 2005; 24:7729–45. 10.1038/sj.onc.1209046 16299533

[18] Freije A, Molinuevo R, Ceballos L, Cagigas M, Alonso-Lecue P, Rodriguez R, Menendez P, Aberdam D, De Diego E, Gandarillas A. Inactivation of p53 in Human Keratinocytes Leads to Squamous Differentiation and Shedding via Replication Stress and Mitotic Slippage. Cell Rep. 2014; 9:1349–60. 10.1016/j.celrep.2014.10.012 25453755

[19] de Pedro I, Galán-Vidal J, Freije A, de Diego E, Gandarillas A. p21CIP1 controls the squamous differentiation response to replication stress. Oncogene. 2021; 40:152–62. 10.1038/s41388-020-01520-8 33097856

[20] Coppé JP, Patil CK, Rodier F, Sun Y, Muñoz DP, Goldstein J, Nelson PS, Desprez PY, Campisi J. Senescence-associated secretory phenotypes reveal cell-nonautonomous functions of oncogenic RAS and the p53 tumor suppressor. PLoS Biol. 2008; 6:2853–68. 10.1371/journal.pbio.0060301 19053174 PMC2592359

[21] Childs BG, Gluscevic M, Baker DJ, Laberge RM, Marquess D, Dananberg J, van Deursen JM. Senescent cells: an emerging target for diseases of ageing. Nat Rev Drug Discov. 2017; 16:718–35. 10.1038/nrd.2017.116 28729727 PMC5942225

[22] Wang AS, Dreesen O. Biomarkers of Cellular Senescence and Skin Aging. Front Genet. 2018; 9:247. 10.3389/fgene.2018.00247 30190724 PMC6115505

[23] Zhang J, Yu H, Man MQ, Hu L. Aging in the dermis: Fibroblast senescence and its significance. Aging Cell. 2024; 23:e14054. 10.1111/acel.14054 38040661 PMC10861215

[24] Pająk J, Nowicka D, Szepietowski JC. Inflammaging and Immunosenescence as Part of Skin Aging-A Narrative Review. Int J Mol Sci. 2023; 24:7784. 10.3390/ijms24097784 37175491 PMC10178737

[25] Kuilman T, Michaloglou C, Vredeveld LCW, Douma S, van Doorn R, Desmet CJ, Aarden LA, Mooi WJ, Peeper DS. Oncogene-induced senescence relayed by an interleukin-dependent inflammatory network. Cell. 2008; 133:1019–31. 10.1016/j.cell.2008.03.039 18555778

[26] Ito Y, Hoare M, Narita M. Spatial and Temporal Control of Senescence. Trends Cell Biol. 2017; 27:820–32. 10.1016/j.tcb.2017.07.004 28822679

